# Influence of sentinel lymph node biopsy on the prognosis of acral melanoma patients^☆^

**DOI:** 10.1016/j.abd.2025.501129

**Published:** 2025-06-18

**Authors:** Thiago Francischetto, Ana Clara Falcão, Adson Santos Neves, Ana Beatriz Lira, Robson Freitas de Moura, Thiago Souza Oliveira Freitas de Moura, Juvandy Antonio Inacio Santos, André Bacellar Costa Lima, Marco Antonio Oliveira Lessa, Jussamara Britos Santos, Alexandre Farias de Albuquerque, Vaner Paulo da Silva Fonseca Pinheiro

**Affiliations:** a Liga Bahiana Contra o Câncer, Hospital Aristides Maltez, Salvador, Bahia, Brazil; b Faculty of Medicine, Universidade Federal da Bahia, Salvador, Bahia, Brazil; c Hospital Santa Izabel, Santa Casa de Misericordia da Bahia, Salvador, Bahia, Brazil

**Keywords:** Biopsy, Melanoma, Prognosis, Sentinel lymph node

## Abstract

**Background:**

Sentinel lymph node (SLN) biopsy in melanoma patients has an important role in staging, prognosis assessment, and treatment definition. Few studies have evaluated its role in the Acral Melanoma (AM) subtype.

**Objective:**

To evaluate the results of SLN biopsy in 79 patients with acral melanoma treated at a single oncological center and compare them with the data described in the literature.

**Methods:**

Between January 2016 and December 2022, the authors analyzed all patients with AM who underwent SLN biopsy in a single institute. The authors analyzed the epidemiological, clinical and histopathological data. Overall Survival (OS) and Disease-Free Survival (DFS) curves were estimated using the Kaplan-Meier method. Multivariate analyses were conducted using the Cox regression model.

**Results:**

During the period, the authors analyzed 79 cases. The mean age was 60 years and median thickness was 4.5 mm and 67.1% had ulceration. SLN was positive in 27 patients (34.2%). The estimated OS and DFS were 67.7% and 45.2%. OS was better in the negative SLN group compared to the positive SLN group (70.9% vs. 53.2%), but without statistical significance (p = 0.08). Estimated DFS for positive SLN was associated with a significantly worse prognosis (33.8 × 46.7%, p = 0.001).

**Study limitations:**

The retrospective nature of the study and the limited number of patients.

**Conclusions:**

The present study has significant implications for determining prognosis. Patients with AM and positive SLN had a worst prognosis compared to those with negative SLN.

## Introduction

Acral lentiginous melanoma is a rare subtype that accounts for 1% to 3% of melanoma cases, primarily affecting the acral skin of the palm, nails, and, especially, the sole of the foot. It is not directly related to sun exposure and is the most common subtype among individuals of African and Asian descent.^1^, ^2^, ^3^ In Brazil, it has a higher incidence than the global average, attributed to the racial diversity of the population. Some studies suggest that acral melanoma is associated with an unfavorable prognosis when compared to other subtypes.^3^, ^4^, ^5^ This may be partly explained by diagnoses at more advanced stages. However, some Asian studies do not confirm these findings and suggest a better prognosis.^6^, ^7^, ^8^

Despite its rarity and specific characteristics, the treatment of acral melanoma follows the same principles as other subtypes. Sentinel lymph node (SLN) biopsy is currently recommended for patients with intermediate-thickness melanomas and has been widely used with low morbidity.^9^, ^10^, ^11^ Since the publication of The Second Multicenter Selective Lymphadenectomy Trial (MSLTII) and Dermatologic Cooperative Oncology Group Trial (DECOG), patients with a positive SLN have been spared of complete lymph node dissection, and with the introduction of new effective treatments, the role of SLN biopsy has been questioned.^12^, ^13^ However, its role has proven important in prognosis assessment, staging melanoma more accurately, and defining complementary treatment strategies. Its therapeutic role is still under debate, but data from MSLTI suggest an improvement in prognosis in patients with thick melanoma and better local control with a reduction in nodal recurrence in patients undergoing SLN biopsy.^14^, ^15^

When analyzing major prospective studies, the majority of patients had the superficial spreading subtype, located on the trunk and extremities, while a smaller number had the acral subtype.^12^, ^13^, ^14^, ^15^ Most studies on acral melanoma are retrospective, involving a limited number of patients, and have been conducted in Asia.^7^, ^8^, ^16^, ^17^, ^18^, ^19^, ^20^, ^21^, ^22^

Due to the lack of data on the use of SLN biopsy in this subgroup of patients and the high proportion of patients with this subtype in Brazil, it is essential to obtain more information about the use of SLN biopsy in this group. Therefore, the aim of this study is to evaluate the results of SLN biopsy in 79 patients with acral melanoma treated at a single oncological center and compare them with the data described in the literature.

## Methods

### Patient selection

Between January 2016 and December 2022, a retrospective cohort of 79 patients with acral melanoma consecutively submitted to SLN biopsy and lymphatic mapping at a single institution was analyzed. The inclusion criteria were patients with confirmed histological diagnosis of acral cutaneous melanoma, absence of lymph node enlargement and Breslow greater than 1 mm or presence of ulceration, Clark IV or V. There were no limits regarding gender, age or skin color.

### SLN biopsy and lymphatic mapping

SLN biopsy and lymphatic mapping were performed by the same team of surgeons. Dynamic lymphoscintigraphy was performed on the morning before surgery with an intra-dermal injection of 1 mCi 99Tcm-phytate. Vital blue dye was injected subdermally around the primary melanoma or biopsy site at the time of surgery. SLNs were localized with a handheld gamma probe intraoperatively, and by visual inspection for blue dye. In vivo and ex vivo counts of the radiolabeled lymph nodes were obtained and compared with nodal bed counts before and after removal. All “hot” and “stained” lymph nodes were considered as SLNs and excised.

### Pathological assessment and treatment choices

SLNs were not evaluated during perioperative time. The SLNs were processed in the pathology laboratory, stained with Hematoxylin and Eosin (H&E) and with the immunohistochemical markers HMB 45, Melan A, and S100. Complete lymph node dissection was initially scheduled in all positive cases. After the publication of MSLT II e DECOG, a conservative approach was offered to positive SLNs patients.

The micromorphometric classification adopted was based on maximum diameter. Invasion depth from the capsule was also evaluated. Metastatic deposits identified in the SLNs had their larger diameter measured using an optical microscope with an ocular micrometer. The largest value defined the SLN’s tumor burden. If multiple positive SLNs were present, the largest maximum diameter in any of the SLNs was considered. The number of metastatic deposits in each SLN was also evaluated.

### Data collection

Data were retrospectively collected from the Hospital’s medical records and electronic databases. Postoperative follow-up was performed in the melanoma outpatient clinic and consisted of a clinical examination every 3-months and chest radiography every 6-months. Other examinations were performed according to the patient’s needs. The patient's current status was classified as alive without evidence of disease, alive with disease, dead from disease, or dead from other causes.

### Outcomes and statistical analysis

The statistical analyses were performed using the Statistical Package for Social Sciences (Version 18.0; SPSS Inc., Chicago, IL, USA). The Chi-Square test was used to evaluate relationships between categorical variables. Disease-free survival (DFS) and overall survival (OS) were calculated from the date of SLN biopsy to date of first recurrence or death, respectively, censored at the date of last contact if there were no events.

Univariate estimations of survival were performed by the Kaplan-Meier method and compared by using the log-rank test. Multivariate analysis was performed with the Cox proportional-hazards regression model including variables with statistical significance on univariate analysis; p-value of less than 0.05 was considered statistically significant. The study was approved by the Committee on Human Research Publications and Ethics of Santa Izabel Hospital.

## Results

### Demographic and preoperative characteristics

In this study, 79 patients diagnosed with acral melanoma underwent SLN biopsy from 2016 to 2022. The median age was 60-years, ranging from 20- to 92-years, with a slight male predominance (51%). 83.5% were classified as black or mixed-race, and 63.3% had comorbidities. 17.7% of patients had a family history of cancer, with 5.1% having a family history of melanoma, and only 1 patient (1.3%) had a previous diagnosis of melanoma (Table 1).

**Table 1:** Clinical and histopathological characteristics.

	Sample (n = 79)
**Gender**	
Male	40 (50.67)
Female	39 (49.4)
**Mean age**	60.4 (±15.36)
**Comorbidities**	
Yes	29 (36.7)
No	50 (63.3)
**Skin Colour**	
White	12 (15.2)
Nonwhite	67 (84.8)
**Previous melanoma**	
Yes	1 (1.3)
No	78 (98.7)
**Anatomical site**	
Plantar	49 (62.0)
Palmar	7 (8.9)
Subungueal hand	12 (15.2)
Subungueal foot	11 (13.9)
**Diagnostic modality**	
Incisional biopsy	39 (49.4)
Excisional biopsy	37 (46.8)
Punch biopsy	1 (1.3)
Unknown	2 (2.5)
**pT Stage, n (%)**	
Tis	2 (2.5)
T1a	1 (1.3)
T1b	8 (10.1)
T2a	9 (11.4)
T2b	7 (8.9)
T3a	5 (6.3)
T3b	14 (17.7)
	5 (6.3)

The diagnosis was mainly made through incisional biopsy (49.4%) or excisional biopsy (46.8%) followed by unknown (2.5) and punch biopsy (1.3). The preferred location was the sole of the foot (62%), followed by subungeal hand (15.2%), subungeal foot (13.9%) and palmar (8.9%).

### Operative outcomes

All patients underwent surgical resection with curative intent, and amputation was necessary in 19% of patients. The intraoperative surgical margin was greater than 1 cm in 72.1%. All patients had a clinically negative lymph node chain and underwent SLN biopsy. In 100% of cases, patent blue dye was used, and only 2 cases (2.5%) did not undergo preoperative lymphoscintigraphy.

The median number of identified sentinel lymph nodes was 1.75, ranging from 1 to 6, with 82.2% having 1 or 2 sentinel lymph nodes. The preferred drainage chain was the inguinal in 72.2% of the cases. In the anatomopathologic study of the surgical specimen, the Clark level was above III in 87.3%, ulceration was present in 67.1%, regression in 7.6%, and satellitosis in 15.2%. The median Breslow thickness was 4.5 mm, ranging from 0 to 25 mm (Table 2).

**Table 2:** Operative and postoperative outcomes.

	Sample (n = 79)
**Clark, n (%)**	
I	1 (1.3)
II	4 (5.1)
III	5 (6.3)
IV	46 (58.2)
V	18 (22.8)
Unknown	5 (6.3)
**Ulceration, n (%)**	
Yes	53 (67.1)
No	26 (32.9)
**Breslow depth, mm**	
Mean	4.5
Median	3.5
**Surgical Margins, n (%)**	
Positive	3 (3.8)
Negative	76 (96.2)
**Surgical ressection, n (%)**	
Large ressection	64 (81)
Amputation	15 (19)
**Sentinel lymph node, n (%)**	
Positive	27 (34.2)
Negative	52 (65.8)
**Positive sentinel lymph node treatment, n (%)**	
Elective lymphadenectomy	14 (51.8)
Observation	13 (48.2)
**Observation Group, n (%)**	
Recurrence	1 (7.7)
No-recurrence	12 (92.3)
**Non-Sentinel Lymph Node, n (%)**	
Positive	10 (71.4)
Negative	4 (28.6)
**Adjuvant Treatment, n (%)**	
Citotoxic chemotherapy	4 (5.1)
Interferon	4 (4.1)
Radiotherapy	5 (6.3)
None	
**Median 5-year overall survival (%)**	66 (83.5)
**Median 5-year disease-free survival (%)**	67.7%
**Median follow-up (mo)**	45.2%
**Clark, n (%)**	26.8

Sentinel lymph nodes were positive in 27 patients (34.2%). Additionally, 17 patients (21.5%) underwent complete lymphadenectomy of the drainage chain. Among these patients, 14 (82.3%) had lymphadenectomy due to a positive SLN, 2 (11.8%) had lymphadenectomy due to nodal recurrence after negative SLN biopsy, and 1 (5.9%) had lymphadenectomy due to nodal recurrence after conservative treatment with a positive SLN. The median number of lymph nodes dissected was 7.7 (ranging from 4 to 12), and the median number of positive lymph nodes was 1.5 (ranging from 0 to 6).

Analyzing the 27 patients with positive sentinel lymph nodes, 14 (51.8%) underwent elective lymphadenectomy. Among these patients, only 4 (28.6%) had no other positive lymph nodes besides the SLN. In 13 patients with positive SLN (48.2%), a conservative approach was chosen, and only 1 had a nodal recurrence and underwent therapeutic lymphadenectomy. The size of the tumor deposit was described in only 8 patients and the median of the largest diameter was 2.4 mm, ranging from 1 to 5 mm. Capsular infiltration was found in 9 patients (33.3%).

No patient received neoadjuvant treatment, and only 16.5% of them received adjuvant treatment. Cytotoxic chemotherapy was performed in 5.1%, Interferon (INF) in 5.1%, and radiotherapy in 6.3%. None of the patients had access to treatments such as immunotherapy or BRAF inhibitors.

### Mortality and survival

The median follow-up was 26.8-months. The mortality rate was 6.3% (5-cases), and the recurrence rate was 26.6% (21-cases). The most frequent site of recurrence was “in-transit” (8 cases ‒ 40%), followed by distant recurrence in 35% (7-cases), being the lung the most frequent site (15%) of distant recurrence. Local recurrence occurred in 3 cases (15%), and nodal recurrence also occurred in 3 cases (15%) (Table 3).

**Table 3:** Comparison of the sentinel lymph node status with clinical and histopathological characteristics of 79 patients with acral melanoma.

	Negative SLN* (n = 52)	Positive SLN* (n = 27)
**Age, yrs**		
Range (average – SD)	26‒91	20‒92
Median	61.33	55.02
**Sex, n (%)**		
Male	27 (51.9)	13 (48.1)
Female	25 (48.1)	14 (51.9)
**Skin color, n (%)**		
White	7 (13.5)	5 (18.5)
Nonwhite	45 (86.5)	22 (81.5)
**Comorbities, n (%)**		
Yes	18 (34.6)	11 (40.7)
No	34 (65.4)	16 (59.3)
**Anatomical site, n (%)**		
Plantar	33 (63.4)	17 (63)
Palmar	4 (7.7)	2 (7.4)
Subungueal hand	8 (15.4)	4 (14.8)
Subungueal foot	7 (13.5)	4 (14.8)
**Diagnostic modality, n (%)**		
Incisional biopsy	30 (57.7)	9 (33.3)
Excisional biopsy	21 (40.4)	16 (59.3)
Punch biopsy	1 (1.9)	‒
Unknown	‒	2 (7.4)
**Breslow depth, mm**		
Mean	3.638	6.426
Median	2.350	4.5
**pT Stage, n (%)**		
Tis	2 (3.8)	‒
T1a	1 (1.9)	‒
T1b	7 (13.5)	1 (3.7)
T2a	9 (17.3)	‒
T2b	5 (9.6)	2 (7.4)
T3a	4 (7.7)	1 (3.7)
T3b	7 (13.5)	7 (25.9)
T4a	3 (5.8)	2 (7.4)
T4b	14 (26.9)	14 (51.9)
**Clark, n (%)**		
I	1 (1.9)	‒
II	4 (7.7)	‒
III	4 (7.7)	1 (3.7)
IV	31 (59.6)	15 (55.6)
V	8 (15.4)	10 (37)
Unknown	4 (7.7)	1 (3.7)
**Ulceration, n (%)**		
Yes	30 (57.7)	23 (85.2)
No	22 (42.3)	4 (14.8)
**Surgical Margins, n (%)**		
Positive	51 (98.1)	25 (92.6)
Negative	1 (1.9)	2 (7.4)
**Surgical Ressection, n (%)**		
Large resection	39 (75)	25 (92.6)
Amputation	13 (25)	2 (7.4)
**Adjuvant Treatment, n (%)**		
Citotoxic chemotherapy	2 (3.8)	2 (7.4)
Interferon	‒	4 (14.8)
Radiotherapy	‒	5 (18.5)
None	50 (96.2)	16 (59.3)
**Follow-up Status**		
Alive without disease	46 (88.5)	14 (51.9)
Alive with disease	5 (9.6)	9 (33.3)
Death	1 (1.9)	4 (14.8)
**Recurrence Pattern, n (%)**		
Non-recurrence	44 (84.6)	14 (51.9)
Local	3 (5.8)	‒
Regional Lymph Node basis	1 (1.9)	2 (7.4)
In transit	3 (5.8)	5 (18.5)
Distant	1 (1.9)	6 (22.2)

**Figure 1:**
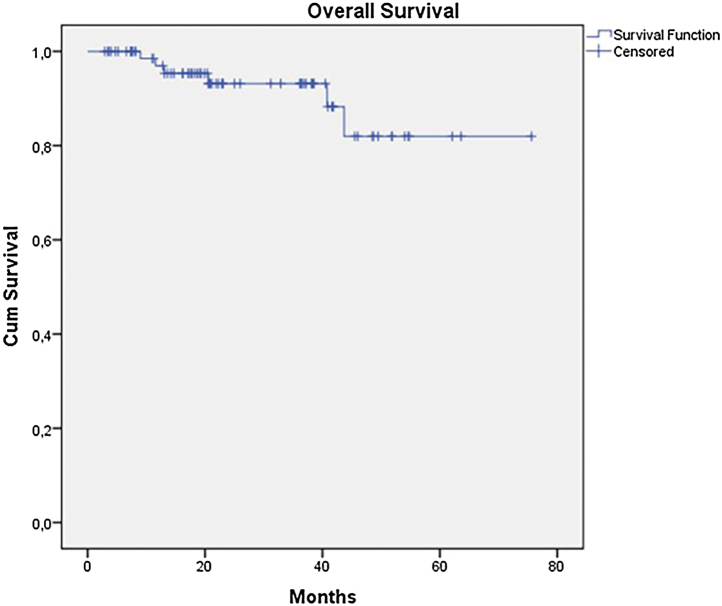
Kaplan-Meier overall survival curves for acral melanoma submitted a sentinel lymph node biopsy.

**Figure 2:**
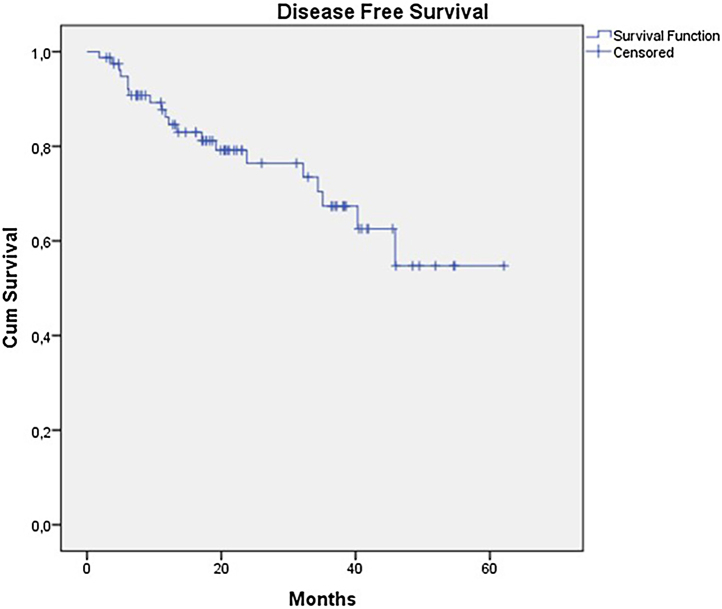
Kaplan-Meier disease free survival curves for acral melanoma submitted a sentinel lymph node biopsy.

**Figure 3:**
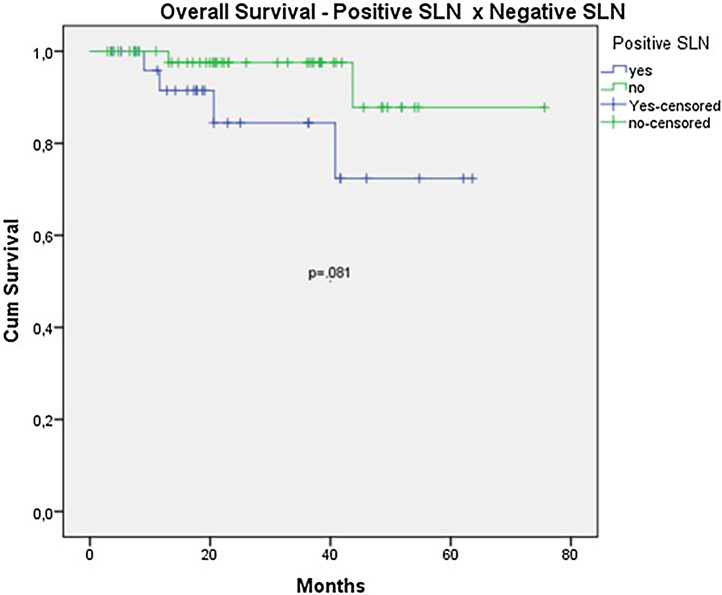
Kaplan-Meier overall survival curves for acral melanoma submitted a sentinel lymph node biopsy by status of sentinel lymph node.

**Figure 4:**
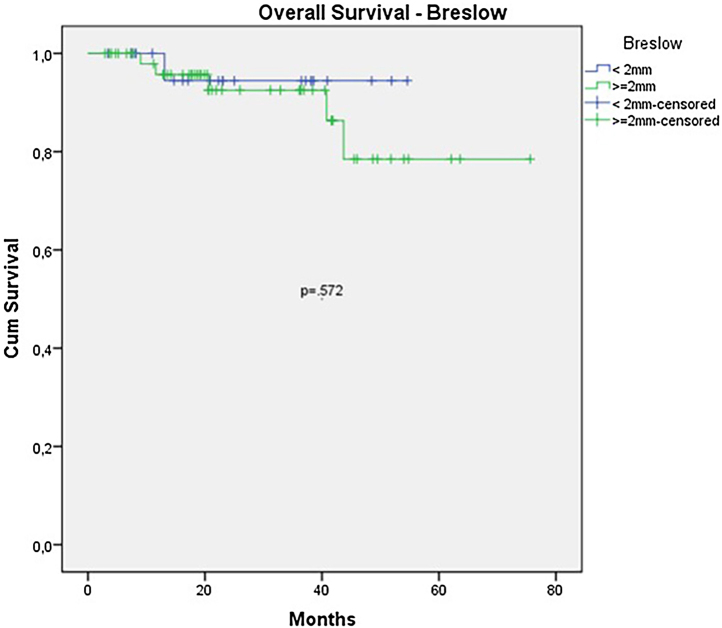
Kaplan-Meier overall survival curves for acral melanoma submitted a sentinel lymph node biopsy by Breslow < 2 mm versus ≥ 2 mm.

**Figure 5:**
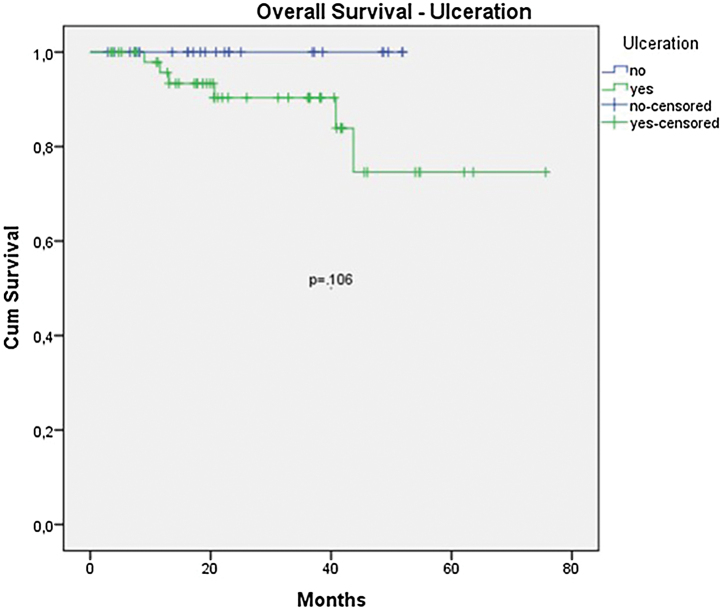
Kaplan-Meier overall survival curves for acral melanoma submitted a sentinel lymph node biopsy by ulceration.

**Figure 6:**
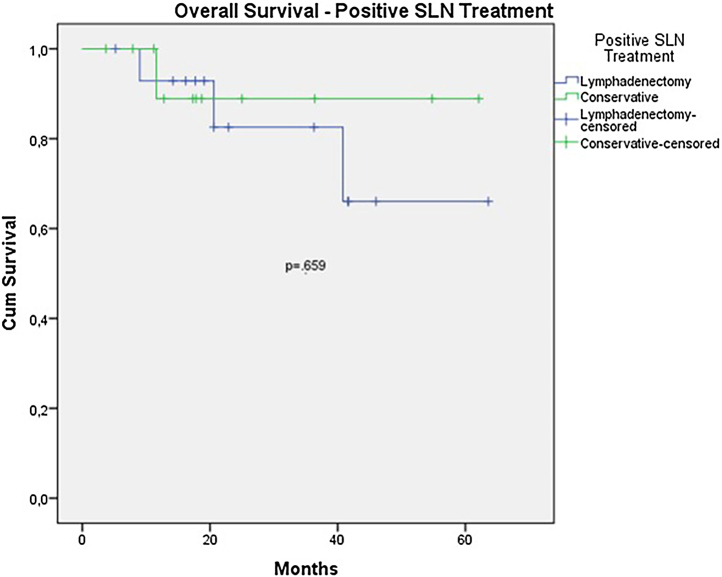
Kaplan-Meier overall survival curves for acral melanoma with positive sentinel lymph node by treatment employed.

The OS for the entire group was 67.7%, and the DFS was 45.2% (Figure 1, Figure 2). In the univariate analysis, OS was better in the negative SLN group compared to the positive SLN group (70.9% vs. 53.2%) (Fig. 3), but without statistical significance (p = 0.081), as was the case in the analysis stratified by Breslow thickness (Fig. 4) (p = 0.572) and ulceration (Fig. 5) (p = 0.106). The OS of patients with positive SLN who were treated conservatively was 56.4%, compared to 51.5% in the group of patients with positive SLN treated with elective lymphadenectomy (p = 0.659) (Fig. 6).

**Figure 7:**
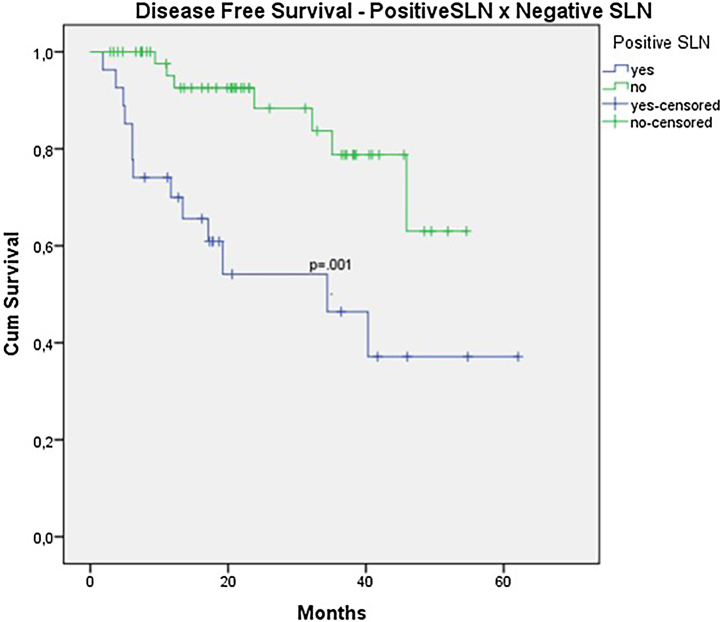
Kaplan-Meier disease free survival curves for acral melanoma submitted a sentinel lymph node biopsy by status of sentinel lymph node.

**Figure 8:**
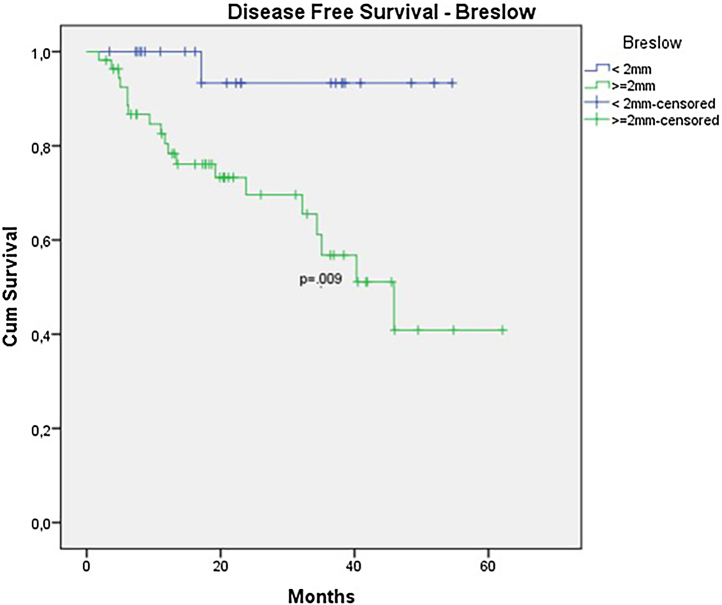
Kaplan-Meier disease free survival curves for acral melanoma submitted a sentinel lymph node biopsy by Breslow < 2 mm versus ≥ 2 mm.

**Figure 9:**
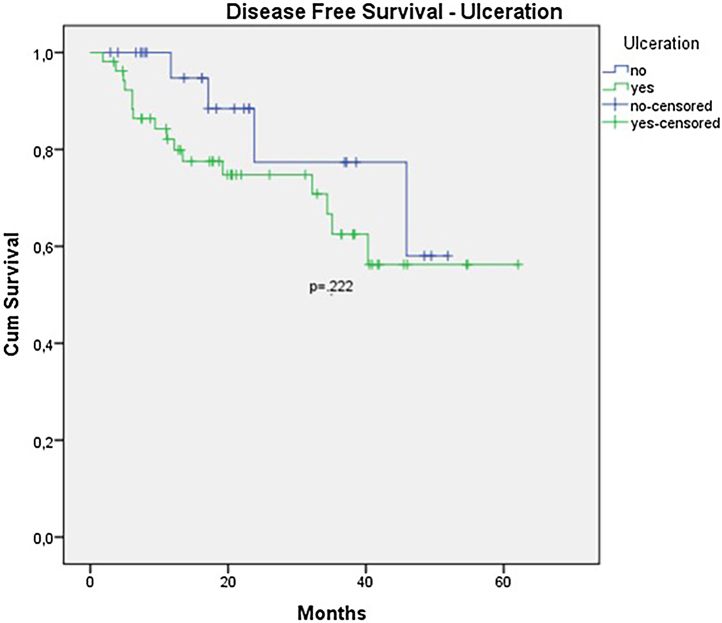
Kaplan-Meier disease free survival curves for acral melanoma submitted a sentinel lymph node biopsy by ulceration.

**Figure 10:**
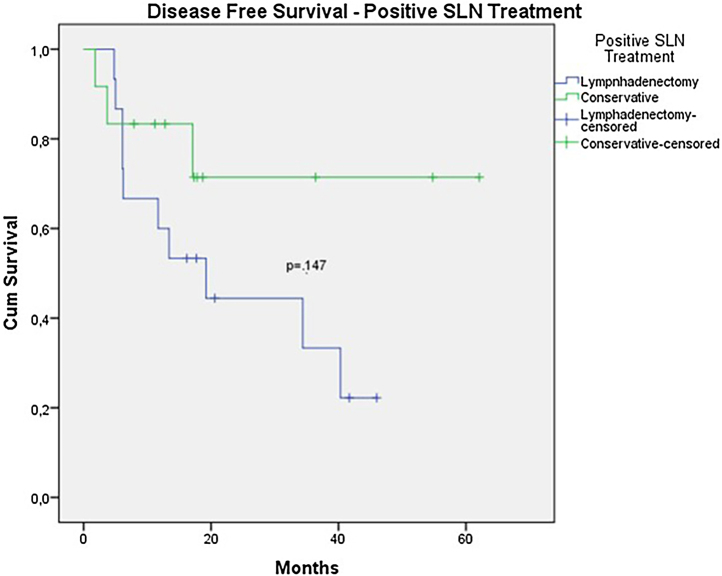
Kaplan-Meier disease free survival curves for acral melanoma with positive sentinel lymph node by treatment employed.

In the univariate analysis of DFS, positive SLN (Fig. 7) and Breslow thickness above 2 mm (Fig. 8) were associated with a significantly worse prognosis (33.8% × 46.7%, p = 0.001 and 40 × 52,1, p = 0.009). The other variables, ulceration (Fig. 9) and the treatment performed in patients with positive SLN (Fig. 10) did not reach statistical significance (p = 0.222, p = 0.147).

In the multivariate analysis, none of the variables showed statistical significance (Breslow thickness, presence of ulceration, regression, age, race, nodal status, tumor location or Clark level) (Table 4, Table 5).

**Table 4:** Factors associated with 5-year overall survival.

	OR	95% CI	p-value
**Age, yrs**	0.967	0.925 – 1.01	0.131
**Sex**	1.003	0.202 – 4.974	0.997
**Skin Color**	1.525	0.175 – 12.279	0.702
**Breslow**	1.021	0.861 – 1.211	0.807
**Clark**	0.661	0.120 – 3.653	0.653
**Ulceration**	35.866	0.023 – 55313.46	0.339
Positive SLN*	0.246	0.045 – 1.351	0.107
Positive SLN* treatment	1.661	0.170 – 16.194	0.662

OR, Odds Ratio; CI, Confidence Interval; SLN*, Sentinel Lymph Node.

**Table 5:** Factors associated with 5-year disease free survival.

	OR	95% CI	p-value
**Age, yrs**	0.985	0.961 – 1.010	0.246
**Sex**	0.993	0.413 – 2.388	0.988
**Skin Color**	2.952	0.670 – 12.997	0.152
**Breslow**	1.117	1.047 – 1.191	0.001
**Clark**	0.000	0.120 – 1.141	0.952
**Ulceration**	1.958	0.652 – 5.882	0.231
Positive SLN*	0.240	0.095 – 0.604	0.002
Positive SLN* treatment	2.514	0.690 – 9.158	0.162

OR, Odds Ratio; CI, Confidence Interval; SLN*, Sentinel Lymph Node.

## Discussion

In this study, the authors retrospectively analyzed the outcome of 79 patients with acral melanoma who underwent SLN biopsy over a 6-year period at a reference center. The present data reveals a patient profile with thicker melanomas (4.5 mm) and a higher rate of ulceration (67.1%), which is above the averages reported in studies like MSLTI, MSLT II, and DECOG.^12^, ^13^, ^14^, ^15^ It also exceeds the study from SEER involving patients with acral melanoma who underwent regional SLN biopsy (median Breslow thickness of 2 mm and 34% had ulceration) and has similar results of a Brazilian study with 66.7% of ulceration and a median Breslow of 5 mm.^16^, ^17^, ^18^, ^19^, ^20^, ^21^, ^22^, ^23^

These data may explain the elevated rate of positive SLN at 34.2% in this study. Most studies on melanoma report positive SLN rates around 20%.^24^, ^25^, ^26^ Studies specifically involving acral melanoma often show divergent results, often due to a limited number of patients and their retrospective nature, with rates ranging from 11.9% to 42.2%.^7^, ^8^, ^16^, ^17^, ^18^, ^19^, ^20^, ^21^, ^22^ A Korean study with only 34 patients showed a positive SLN rate of 41.2%,^17^ while another Korean study, with a larger number of patients (107) treated over a 12-year period, reported a positive SLN rate of 20.6.^18^ A retrospective study using a U.S. database with 753 patients, mostly of Caucasian descent, showed a positive SLN rate of 25.7%,^16^ and a Brazilian study with 201 acral melanoma patients, showed a positive SLN rate of 29.9%.^23^

In all patients, SLNs were correctly identified. This is consistent with the findings of Asian studies involving acral melanoma, which can be attributed to the location of acral lesions on extremities with a more predictable drainage chain. This also explains the high rate of SLN identification using the combined technique of blue dye and lymphoscintigraphy.^7^, ^8^, ^17^, ^18^, ^19^, ^20^, ^21^, ^22^ Some data suggest that the false-negative rate for SLNs in acral melanoma patients could be higher. However, this data is not consistent with other studies,^27^, ^28^, ^29^ and it was not observed in the present study, where the recurrence rate at the drainage site after a negative SLN was only 3.8% (2 cases out of 52), which is lower than that reported in MSLT I 5.5%) and a Korean study (11.8%).^14^, ^18^

The SLN biopsy has proven to be important in assessing patients' prognosis. Nevertheless, the number of patients with acral melanoma has always been limited in prospective Western studies.^12^, ^13^, ^14^, ^15^ Specific studies on acral melanoma are retrospective and originate from Asia. Despite these limitations, seven studies have shown that the presence of SLN metastasis is a significant risk factor correlated with survival rates, especially in terms of DFS.^8^, ^17^, ^18^, ^19^, ^20^, ^21^, ^23^

In this study, an OS of 67.7% was observed in the total group, which is lower than what is typically seen in melanoma studies, ranging around 91.7%, and lower than other retrospective studies involving acral melanoma, which typically report survival rates around 80%.^3^, ^4^ The reasons for this unfavorable prognosis can be explained by the more advanced diagnosis in the patients as the authors can observe in a Brazilian study that has similar characteristics and an even worse prognosis (OS of 44.6% and 38.6% DFS).^23^

When analyzing SLN results, the authors observed a clear trend toward improved survival in patients with negative SLNs (OS 70.9% vs. 53.2% with p = 0.081). However, statistical significance was only achieved in terms of DFS (46.7% vs. 33.9% with p = 0.001). The authors believe that, due to the retrospective nature of the study and the limited number of patients, statistical significance was not reached. Other studies focusing exclusively on acral melanoma have also shown similar results. In a study involving acral melanoma patients in the United States, melanoma-specific survival was 88.5% in the negative SLN group, compared to 58.9% in the positive SLN group.^16^ In six Asian studies, including three Japanese, one Chinese, and two Korean studies, positive SLN correlated with worst survival, especially in terms of DFS.^8^, ^17^, ^18^, ^19^, ^20^, ^21^

Since the publication of DECOG and MSLT II, conservative treatment after a positive SLN has been recommended.^12^, ^13^ In the present study, only half of the patients (48%) followed this recommendation, largely because data supporting this approach emerged during the course of the study. Additionally, the authors are treating a population with more advanced lesions, with substantial tumor deposits in the SLN, far beyond that was described in patients in the DECOG and MSLT II studies. This also justifies why Only 4 out of 14 patients who underwent elective lymphadenectomy after a positive SLN did not have any other positive lymph nodes besides the SLN.

Nevertheless, despite all these data, in the analysis of subgroups with positive SLNs, OS, and DFS were worse in the group that underwent elective lymphadenectomy after a positive SLN. However, this difference did not reach statistical significance (OS 51.5% vs. 56.5% with p = 0.659; DFS 23.8% vs. 46.9% with p = 0.147). In this group, where the conservative approach was adopted, Only 1 patient underwent lymphadenectomy due to local recurrence. The mortality rate was 9.3% in the group that underwent conservative treatment and 20% in the group that underwent lymphadenectomy after a positive SLN. These data may suggest that lymphadenectomy was indicated in patients with a larger tumor volume, but it also suggests that conservative treatment after a positive SLN could be offered in acral melanoma patients.

## Conclusion

In this study, the SLN biopsy in acral melanoma patients demonstrated high accuracy in identifying sentinel lymph nodes and had significant implications for determining prognosis. Patients with acral melanoma and positive sentinel lymph nodes had a worse prognosis compared to those with negative sentinel lymph nodes. Patients with positive SLN biopsy who underwent lymphadenectomy showed a prognosis like those treated expectantly, suggesting that this approach could also be recommended for this subgroup of patients. However, due to the retrospective nature of the study and the limited number of patients, each patient should be assessed individually.

## Financial support

None declared.

## Authors’ contributions

Thiago Francischetto: Conceptualization and design of the study; original draft; critical review of the literature; final approval of the final version of the manuscript.

Ana Clara Falcão: Data collection; statistical analysis.

Adson Santos Neves: Original draft; obtaining, analyzing and data interpretation.

Ana Beatriz Lira: Data collection; statistical analysis.

Robson Freitas de Moura: Obtaining, analyzing and data interpretation.

Thiago Souza Oliveira Freitas de Moura: Original draft; obtaining, analyzing and data interpretation.

Juvandy Antonio Inacio Santos: Original draft; obtaining, analyzing and data interpretation.

André Bacellar Costa Lima: Original draft; obtaining, analyzing and data interpretation.

Marco Antonio Oliveira Lessa: Original draft; obtaining, analyzing and data interpretation.

Jussamara Britos Santos: Original draft; obtaining, analyzing and data interpretation.

Alexandre Farias de Albuquerque: Original draft; obtaining, analyzing and data interpretation.

Vaner Paulo da Silva Fonseca Pinheiro: Statistical analysis; obtaining, analyzing and data interpretation; Final approval of the final version of the manuscript.

## Conflicts of interest

None declared.
